# Positive identity predicts psychological wellbeing in Chilean youth: A double-mediation model

**DOI:** 10.3389/fpsyg.2022.999364

**Published:** 2022-11-24

**Authors:** Pablo A. Pérez-Díaz, Sergio Nuno-Vasquez, Matheus França Perazzo, Nora Wiium

**Affiliations:** ^1^Institute of Psychology, Sede Puerto Montt, Austral University of Chile, Puerto Montt, Chile; ^2^Faculty of Psychology, San Sebastián University, Santiago, Chile; ^3^Department of Oral Health, Dental School, Universidade Federal de Goiás, Goiânia, Goiás, Brazil; ^4^Faculty of Psychology, University of Bergen, Bergen, Hordaland, Norway

**Keywords:** positive youth development, positive identity, psychological wellbeing, Chilean adolescents, five Cs model

## Abstract

Positive youth development (PYD) allows the youth to be comprehended from their potential, strengths and assets, in contrast to the traditional deficit view that focuses on their weaknesses. The PYD model promotes constructive behaviours in youth by highlighting the positive attributes usually found during the transition from childhood to adulthood to achieve healthy and optimal development in later life. Overall, PYD comprises five key competence (5C), the flourishing models and forty developmental assets. In the present study, a structural equation model is tested with the Chilean dataset of the PYD project on the premise that Positive Identity is the core internal developmental asset explaining Psychological wellbeing and that Confidence and Character are mediators of the relationship between Positive Identity and Psychological Wellbeing. The sample comprised 261 participants (*n*_Women_ = 189, *n*_Men_ = 72), *Mean*_Age_ = 22 years old, who were approached by an online survey uploaded to Qualtrics. The measures of the study included: The Developmental assets Scale, the Short-form of the Five Cs included in the PYD and the Mental Health Continuum Short-Form. The results indicated a good model fit (*β* = 1.74, *Z*_total_ = 10.63, *χ*^2^ = 424.95, *df* = 277, χ^2^/*df* = 1.53, *p* < 0.001, Robust *CFI* = 0.945, Robust *RMSEA* = 0.049, 90% *CI* (0.040, 0.058), *AIC* = 17689.91, *saBIC* = 17719.08 and *SRMR* = 0.061), highlighting the relevance of studying Latin-American adolescents and young ‘s wellbeing in times of COVID-19, as the participants’ Positive Identity significantly predicted their Psychological Wellbeing, and simultaneously, this relationship was mediated by both their level of Confidence and Character.

## Introduction

Positive youth development (PYD) allows the youth to be understood from their potentialities and external and internal resources. The previous is in opposition to the conventional view emphasised from the second half of the XX century, focusing on what adolescents should not be doing (e.g. Catalano et al., 2004), and fundamentally understanding and treating psychopathology rather than comprehending and fostering developmental thriving (Benson, 2007). Instead, the PYD paradigm promotes constructive behaviours in youth by highlighting the positive attributes usually found during the transition from childhood to adulthood. Indeed, adolescence comprises profound physical, psychological and social growth (Geldhof et al., 2015), all crucial for personality development and healthy psychological and social functioning in later life.

Overall, the PYD paradigm comprises five to seven key competence (5Cs, 6Cs or 7Cs, Lerner et al., 2005; Geldhof et al., 2015; Dimitrova et al., 2021), the development assets theory (Benson, 2007; Scales, 2011) and the flourishing model (Ryff, 1989; Ryff and Keyes, 1995; Keyes, 2005, 2007). Dimitrova et al. (2021) posited that the Cs and the developmental assets are the most influential PYD frameworks, which are usually considered separately in the literature rather than integrated into a single conceptual representation. These models have consistently explained the variance in youth thriving across several behavioural criteria (Lerner et al., 2015). For instance, through wellbeing measures, as included in the flourishing model (Keyes, 2002). However, the Cs model has found more empirical support (Geldhof et al., 2015), and it has been more widely implemented in research and intervention programmes for youth (Geldhof et al., 2014).

### The developmental assets framework

According to Benson (1990, 2007), the developmental assets represent a theoretical construct encompassing a broad range of environmental and interpersonal strengths which predict several academic, psychological, social and health outcomes. The theory comprises 40 elements, which can be split into internal and external assets and is rooted in the large metatheory of developmental system theory (e.g. Ford and Lerner, 1992), which conceives human development as a combination of internal and contextual processes. The 20 internal assets comprise skills, competence and commitments, whereas the 20 external assets include socialising systems’ environmental, contextual and relational features (Benson, 2007). As highlighted by Benson, the theory relies on the belief that young people select a subset of resources that provide advantages for their own life goals.

The internal assets include four factors: commitment to learning, positive values, social competence and Positive Identity. These resources can be further decomposed into 20 facets. Therefore, commitment to learning comprises the following facets: achievement motivation, school engagement, homework, bonding to school and reading for pleasure. Positive values engulfs the facets of caring, equality and social justice, integrity, honesty, responsibility and restraint. Social competence comprehends planning and decision-making, interpersonal competence, cultural competence, resistance skills and peaceful conflict resolution. Finally, Positive Identity includes the facets of personal power, self-esteem, sense of purpose and positive view of the personal future. Moreover, the Positive Identity construct has been defined as “a sense of control and purpose, as well as recognition of own strengths and potentials, including personal power, self-esteem, and positive outlook” (Dimitrova and Wiium, 2021, p. 5).

The external assets include four factors: support, empowerment, boundaries and expectations and constructive use of time. All of which can be further decomposed into 20 facets. Hence, support includes family support, positive family communication, other adult relationships, caring neighbourhood, caring school climate and parent involvement in schooling. Empowerment engulfs community values youth, youth as resources, service to others, and safety. Boundaries and expectations comprise family boundaries, school boundaries, neighbourhood boundaries, adult role models, positive peer influence and high expectations. Finally, constructive use of time includes creative activities, youth programmes, religious community and time at home.

### The five competence framework (5Cs)

According to Bradshaw and Guerra (2008), the five Cs provide a shared nosology for investigating wellbeing indicators in youth. Initially conceptualised by Little (1993), the latent construct structure of the Cs framework comprehended four key factors, namely: competence, Confidence, Connection and Character, from which other prominent authors expanded the framework by incorporating the fifth C denominated Caring (e.g. National Research Council and Institute of Medicine, 2002; Lerner, 2004). Scholars (Roth and Brooks-Gunn, 2003; Lerner, 2004) have posited that Competence alludes to the positive view of one’s actions in specific areas, such as academic, cognitive and vocational, and that Confidence refers to an internal sense of overall positive self-worth and efficacy. In addition, Connection indicates positive bonds with people and organisations in which there are bidirectional exchanges between youth and society, such as family, school and the community. Moreover, Character denotes respect for societal and cultural norms, in addition to the internalisation of standards for socially acceptable behaviour and a sense of correctness and integrity (Lerner et al., 2005). Finally, Caring denotes a sense of sympathy and empathy for others (Lerner, 2004).

On top of these competence, a sixth C (Contribution) has been proposed (Lerner et al., 2003), which stands for behaviours emanating from the 5Cs affecting individual’s self, family, community and civil expanded society (Lerner, 2004). Moreover, a new theoretical and empirical development has been recently introduced by Dimitrova et al. (2021). According to these authors, incorporating a seventh C (Creativity) provides incremental validity to the framework. Creativity stands for a novel-original and useful-adaptative problem-solving ability akin to the specific social and cultural environment in which youth develop. Dimitrova et al. emphasised that conceptually, creativity serves as a bridge between the Cs framework and the developmental assets theory as to the measurement of PYD.

### The flourishing model in emerging adults

As posited by Keyes (2002), half of the adult population remains free of serious mental illnesses during their lifespan, and around 90% are free of major depression each year which illustrates the importance of studying positive functioning instead of psychopathology in cross-cultural research. The flourishing model is based on several wellbeing measures mostly treated by researchers as outcomes of thriving, although these flourishing variables have been conceptualised as independent variables by scholars (Ryff and Keyes, 1995; Keyes, 2005). In either case, they represent a cornerstone in the measurement of positive attitudes, which ranges from physical wellbeing, passing through subjective, social and psychological wellbeing, to eudaimonic wellbeing. Indeed, this progression represents the evolution of the conceptualisation of flourishing in the literature, as early theoretical developments and measures on positive outcomes deviated less from concrete psychopathological criteria than more ambitious and recent developments, such as eudaimonic measures Waterman et al. (2010)

One of the most validated models for comprehending and measuring wellbeing is the mental health continuum paradigm proposed by Keyes (2002). This theory advances on extensive previous developments on subjective wellbeing, a construct that targets happiness and life satisfaction through self-report measures (Conceição and Bandura, 2008), and that comprises individuals’ perceptions and evaluations of their own life based on emotional, psychological and social functioning (Keyes and Waterman, 2003). For Keyes (2002), emotional wellbeing comprises the presence or absence of positive feelings about being alive, psychological wellbeing represents more private and personal criteria for assessing overall functioning, whereas social wellbeing refers to the more public and social criteria by which people appraise their functioning.

All of these wellbeing domains theoretically contribute to either languishing or flourishing in life, as mental health is holistically conceived within the continuum, as individuals are expected to be free of psychopathology, thrive across these domains and consequently experience a lower risk of configuring any illnesses (Keyes, 2005). In spite of the tenable orthogonality pertaining each of these types of wellbeing (Keyes, 2005), there is agreement in the literature that they are strongly correlated and that they mostly share the same factorial space, contributing all to positive psychological functioning (Keyes, 2005; Waterman et al., 2010), as conceptualised by the flourishing model. Keyes (2005) provided support to the stance that measures of mental health (i.e. emotional wellbeing, psychological wellbeing and social wellbeing) and those of mental illness (i.e. panic disorder, major depressive disorder, generalised anxiety and alcohol dependence) constitute two separated correlated axes.

### Aims of the study

There is a thrust in academia to fill the existent gaps in the PYD literature, with attention to methodological, theory-driven, inclusive and novel research across related disciplines (Psychology, public health, family studies and public policy) and populations, especially those neglected in the English peer-review scholarship. In this regard, the cross-national project on PYD (CN-PYD) aims to disseminate and equally consider scholarship from a wide range of researchers and countries in the world (Sheik, 2021), as it is the umbrella under which the current research was developed. To provide context, the amount of research in Latin America is still incipient and mostly circumscribed to Peruvian, Colombian and Mexican populations (i.e. Domínguez Espinosa et al., 2021; Manrique-Millones et al., 2021), which does not allow for full generalisation in other culturally distant Latin-American countries, such as Argentina, Brazil and Chile (Ziegler and Bensch, 2013; Greiff and Iliescu, 2017). The current study is aimed at initially filling this gap regarding Chilean Youth.

As Catalano et al. (2019) posited, the application of the PYD paradigm fits perfectly into Latin-American countries, with the developmental assets and the 5Cs in front and state wellbeing measures as criteria, where young people experience significant rates of violence, crime, mental health and behavioural problems in a scarcely resourceful context and more so after COVID pandemic. In this scenario, the relationship between risky behaviours and models to prevent and reduce the occurrence of risky behaviours is of particular relevance, which is essential not only to emerging adults’ wellbeing but also to the long-term welfare and prosperity of the region (Manrique-Millones et al., 2021).

In the present study, a double-mediation structural equation model (SEM) is tested with the first wave of the Chilean PYD project on the following premises: (1) Positive Identity is the core internal developmental asset explaining Psychological Wellbeing (Hypothesis one, namely, H1), and that (2) Confidence and Character are mediators of the relationship between Positive Identity and the dependent variable Psychological Wellbeing (Hypotheses two and three respectively, namely, H2 and H3). Hence, the direct and indirect role of Positive Identify on Psychological Wellbeing will be explored through two salient competence from the 5Cs: Confidence and Character. As highlighted by scholars, PYD modelling is still in current development (Dimitrova et al., 2021). Therefore, there is a need for further theoretical and empirical developments which allow accurately targeting indicators of youth wellbeing (Koller and Verma, 2017), especially through well-articulated and theoretically supported structural analyses (Ryff and Keyes, 1995).

## Materials and methods

### Participants

The sample comprised 261 Chilean juveniles (*n_Women_* = 189, *n_Men_* = 72, *Mean_Age_* = 21.87, *SD_Age_ =* 3.14) who were approached by convenience sampling through an online survey uploaded to Qualtrics. The data collection extended from April to November 2021. The questionnaire was disseminated through a single link by the authors of the study and two undergraduate dissertation teams under the supervision of the principal researcher. The study obtained ethical clearance through the University of Bergen, Norway, dated 24 June 2019, with reference number 612969. In addition, local ethics approval was obtained in Chile on 25 June 2020, from the Vice-rectory of Research, Development and Artistic Creation (VIDCA)-Ethics and Bioethics Committee of the Austral University of Chile and their subcommittee on Bioethics in Human Research. The dataset used in this research has been made freely available online at: https://data.mendeley.com/datasets/76nwjf62kk.

### Measures

#### The developmental assets profile

The developmental assets were measured with The Developmental Assets Profile (DAP; Benson, 2007). The questionnaire comprises 58 items, on a 5-point Likert response scale, across external assets (i.e. Support, empowerment, expectations and boundaries, constructive use of time) and internal assets (i.e. commitment to learning, positive values, social competence and positive identity). The DAP has been implemented across diverse cultural and language populations, encompassing more than 25,000 adolescents and emerging adults aged 9–31 (see Scales et al., 2017). In the current study, all scales showed acceptable to high internal consistency, except for constructive use of time: Support (*α* = 0.78), empowerment (*α* = 0.72), expectations and boundaries (*α* = 0.79) constructive use of time (*α* = 0.40), commitment to learning (*α* = 0.81), positive values (*α* = 0.79), social competence (*α* = 0.75) and positive identity (*α* = 0.84). Only positive Identity was used in the analyses of the present study.

#### The PYD short-form

The 5Cs were measured with the PYD Short-Form (PYD-SF; Geldhof et al., 2014). The questionnaire comprises 34 items, on a 5-point Likert response scale, across five scales (i.e. Competence, Confidence, Character, Caring and Connection). All of these scales displayed acceptable to high internal consistency scores: Competence (*α* = 0.71), Confidence (*α* = 0.87), Character (*α* = 0.64), Care (*α* = 0.79) and Connection (*α* = 0.74), with the lowest Character and the highest Confidence, being the two Cs included in the analyses of the current research. The Developmental Assets Profile and the PYD Short-Form were translated from English to Latin-American Spanish and back-translated to assure linguistic equivalence in previous research conducted by Manrique-Millones et al. (2021) as part of the ongoing cross-cultural study on PYD (Wiium and Dimitrova, 2019).

#### The mental health continuum short-form

The wellbeing measures were assessed through the Spanish Version of the MHC-SF (Echeverría et al., 2017), which is a self-reported 14-item questionnaire on a 6-point Likert response scale originally designed by Keyes (2002); Keyes et al. (2008) to assess emotional (3 items), psychological (6 items) and social (5 items) wellbeing during the last month. All these scales displayed high internal consistency scores: Emotional Wellbeing (*α* = 0.83), Psychological Wellbeing (*α* = 0.89), Social Wellbeing (*α* = 0.76) and the overall score of Wellbeing (*α* = 0.91). Only Psychological Wellbeing was utilised in the analyses of the current research.

### Design and procedure

The study design is cross-sectional. According to Tarka (2018), Structural Equation Modelling refers to a set of equations in which theoretical assumptions are tested on meaningful observable variables (i.e. measurement model) and latent variables (i.e. measurement model). SEM is a sophisticated statistical method for testing complex causal hypotheses from associations (i.e. structure) among observable variables, combining factor analysis, path analysis and multiple regression analysis (Vogt, 2005).

Moreover, SEM analysis can be conducted to study either specific effects or a global model (Agler and De Boeck, 2017), where the former focuses more on specific paths or effects, and the latter has a stronger interest in testing a global model with all the variables of interest and evaluating it on salient criteria. In the present study, we have embraced a global model approach to SEM.

Several standard preliminary analyses were conducted to test the suitability of the data for SEM. Firstly, the measurement model from each implemented scale was tested through CFA. Moreover, the direct relationship between the variables without mediation was tested through SEM, in which the measurement model corresponded to the item level of Positive Identity, Confidence, Character and Psychological Wellbeing, whereas the structural model comprised Confidence and Character, latent variables that were regressed on Positive Identity.

Direct and indirect effects were tested separately for each of the mediators. Lastly, two-way mediation was analysed through an integrated double-mediation model, which will be referred to as the basic model or Model 1 in the manuscript. A second model (Model 2) was assessed after conducting modification indices, in which theoretically (i.e. same construct) and linguistically supported (i.e. B61: I feel my life has a purpose and C35: Your life has a sense of direction or meaning and between B60: I think about my purpose in life and C35: Your life has a sense of direction or meaning) item correlations were included.

**Figure 1:**
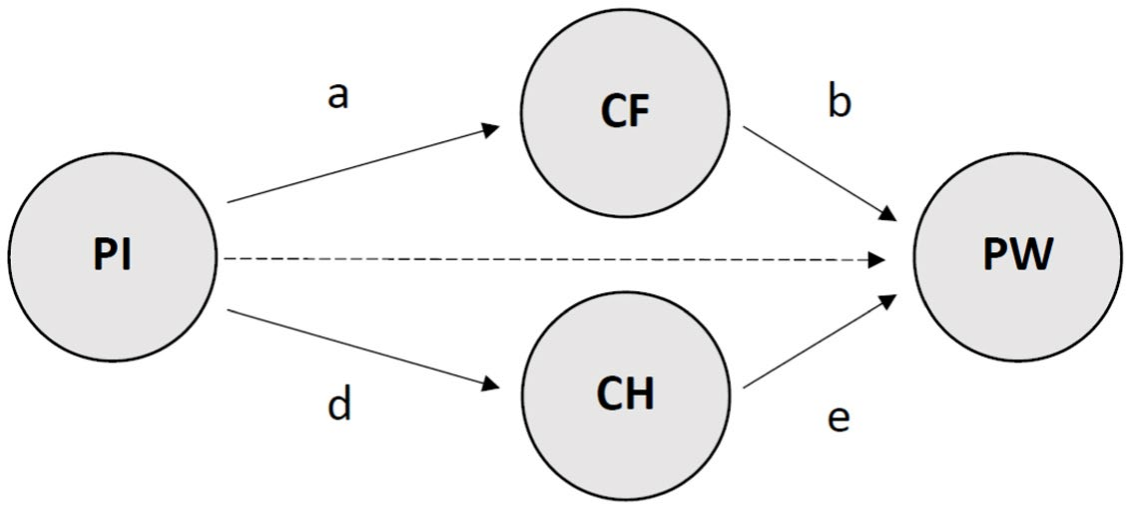
Conceptualisation of the model tested in the research. Direct hypothesised relationships are depicted in solid arrows. The indirect hypothesised relationship is illustrated through a dashed arrow. PI, positive identity; CF, confidence; CH, character; PW, psychological wellbeing.

Finally, a third model (Model 3) included further item correlatedness were allowed between the mediators underlying the same PYD construct (i.e. Confidence and Character), one additional association between two indicators of Positive Identity (i.e. B61: I feel my life has a purpose and B55: I feel that I have the control of my life and future), and one between two indicators of Psychological Wellbeing (i.e. C31: I am good at managing the responsibilities of your daily life and C32: I have warm and trusting relationships with others), which resulted in the final and most accurate depiction of the structural patterns of the research. An illustration of the SEM conceptualisation examined in the study is provided in Figure 1, whilst Table 1 depicts the correlation matrix from the variables included in it. In all the analyses, we evaluated model fit through MLR estimations (maximum likelihood with robust standard errors) following the recommendations by Hu and Bentler (1999), as these authors suggested that Root Mean Square Error of Approximation (RMSEA) values lower than 0.06, Standardised Root Mean Squared Residual (SRMR) under 0.05 and CFI/TLI values close or larger than 0.95 indicate good model fit to the data, reflecting overall the least sum of Type I and Type II error rates with simulated data.

**Table 1:** Correlation matrix of the studied variables.

	PI	CF	CH	PW
PI	1	0.674***	0.330***	0.725***
CF	0.674***	1	0.259***	0.691***
CH	0.330***	0.259***	1	0.372***
PW	0.725***	0.691***	0.372***	1

***Correlation is significant at the 0.001 level (2-tailed).

Moreover, multicollinearity diagnostics were conducted, after which it was determined that multicollinearity between the exogenous variables (Positive Identity, Confidence and Character) was not an issue. More specifically, VIF (Variance Inflation Factor) scores were in the range of 1.125 to 1.925 (*cf.* values above 5 reveal serious multicollinearity, Hair et al., 2011), whilst tolerances were in the range of 0.52 to 0.89 (*cf.* values closer to 0 indicate low tolerance which translates as high multicollinearity, whereas values closer to 1 reveal high tolerance which suggests low multicollinearity, Hair et al., 2014). Consequently, tolerances values below 0.20 are of concern (Hair et al., 2011). In a nutshell, VIF and tolerance scores reflect low multicollinearity for the exogenous variables included in the model. Table 2 provides multicollinearity diagnostics in full. The main analyses were conducted in R Studio build 492 with R build 4.0.4 through the lavaan package (Rosseel, 2012).

**Table 2:** Multicollinearity diagnostics.

	*Beta*	*t*	*Tolerance*	*VIF*
Intercept		−3.156**		
PI	0.437	8.156***	0.520	1,925
CF	0.361	6.898***	0.544	1,839
CH	0.134	3.275**	0.889	1,125

PI, positive identity; CF, confidence; CH, character. Psychological wellbeing (PW) is the dependent variable. **p < 0.01, ***p < 0.001. Standardised coefficients and two-tailed *p*-value of are reported. VIF, variance inflation factor.

## Results

**Figure 2:**
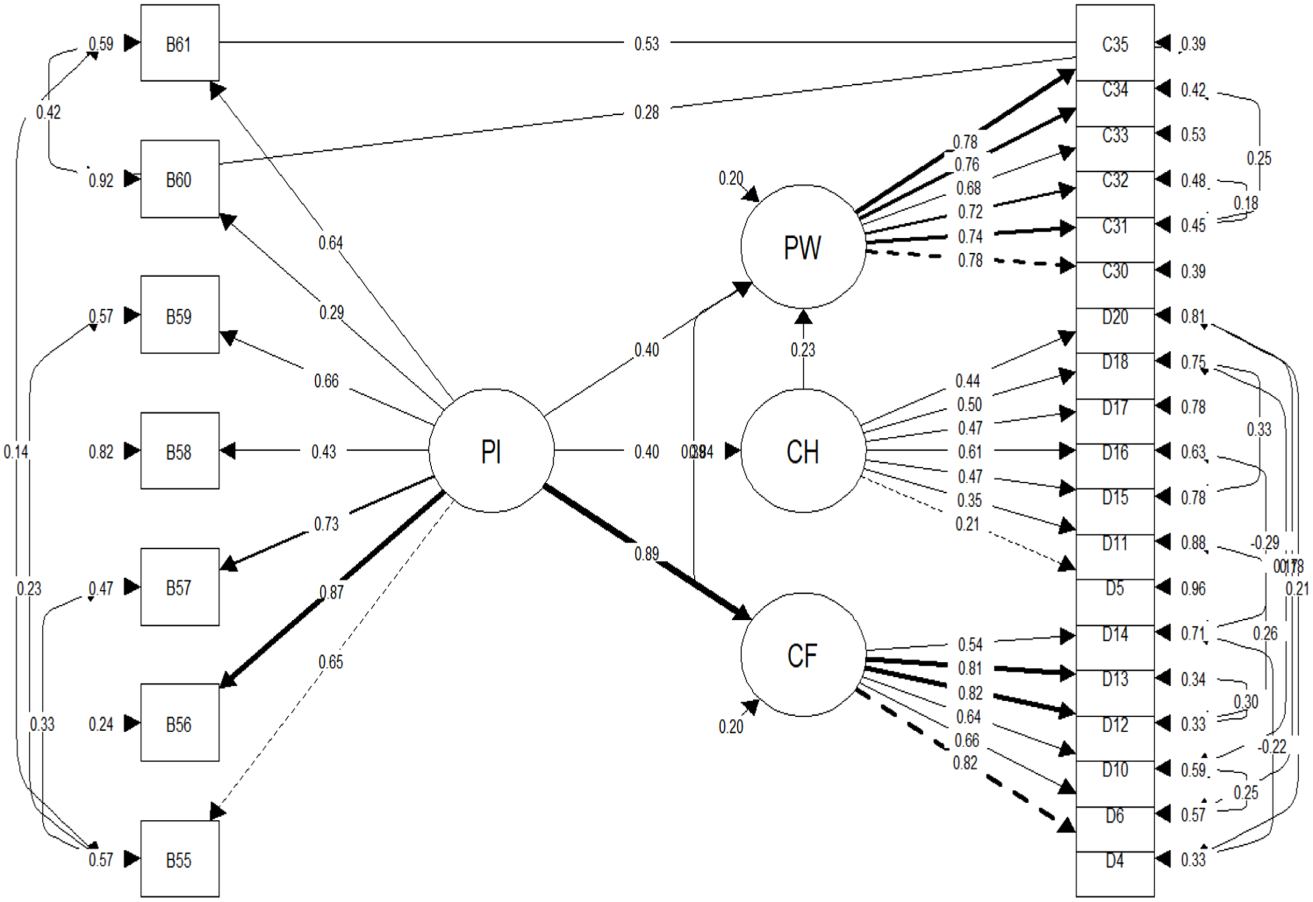
Double mediation model with the introduction of item correlatedness. Item correlatedness is depicted to the left and right of the indicators (items). The item correlatedness mostly correspond to the facet level of PI (personal power, self-esteem, sense of purpose, and positive view of personal future), confidence, and character. Only standardised latent variables values are reported. PI, positive identity; CF, confidence; CH, character; PW, psychological wellbeing.

The basic model (Model 1) without item uniqueness indicated a promising model fit, although the character regression path was not statistically significant. In this model, Positive Identity explained 62.7% of the variance of Confidence, 13.4% of Character and 76.4% of Psychological Wellbeing. Therefore, item correlatedness was introduced in Model 2 due to conducting modification indices, after which the character regression path became statistically significant. In this model, Positive Identity explained 73.6% of the variance of Confidence, 16.7% of Character and 78.7% of Psychological Wellbeing. In Model 3, further theoretically supported item correlation was introduced following model 2 and additional modification indices, after which the model improved significantly to be considered “good” as to current standards (i.e. CFI ≥ 0.95 and all other fit indices between the expected thresholds; Hu and Bentler, 1999), although the Character path returned non-significant. In this model, Positive Identity explained 79.7% of the variance of Confidence, 15.9% of Character and 79.5% of Psychological Wellbeing. This modelling progression is informed in Table 3, where fit indices for each model and Chi-squared changes between the models are presented in full. Moreover, Table 4 portrays the estimated mediation paths through SEM, as obtained in the lavaan package. Finally, Figure 2 illustrates the patterns obtained from the final double-mediation model (Model 3).

**Table 3:** Positive identity modelling tested through SEM with confidence and character as mediators in Chilean youth.

Models	*χ* ^2^	*df*	CFI	RMSEA	RMSEALb	RMSEAUb	AIC	saBIC	SRMR
Basic double-mediation model without M.I.	728.58	319	0.850	0.076	0.069	0.084	18660.93	18684.18	0.073
Double-mediation model with M.I.	468.21	283	0.931	0.054	0.045	0.063	17728.35	17755.15	0.063
Double-mediation model with additional M.I.	424.95	277	0.945	0.049	0.040	0.058	17689.91	17719.08	0.061
*χ*^2^∆ between Models 1 and 2	269,38***	36.00							
*χ*^2^∆ between Models 2 and 3	43,26***	6.00							

*N* = 261. *χ*^2^, Chi-squared; *df*, degrees of freedom; CFI, Comparative Fit Index; RMSEA, Root Mean Square Error of Approximation; RMSEALb, RMSEA Lower bound; RMSEAUb, Upper bound; AIC, Akaike Information Criterion; saBIC, Sample-sized adjusted Bayesian Information Criterion; SRMR, Standardised Root Mean Residual; *χ*^2^∆, Chi-squared changes between tested models. Scaled Chi-squared difference test Satorra-Bentler was implemented. Robust fit test statistics are reported. ***p < 0.001.

**Table 4:** Positive identity double-mediation estimated paths through SEM in Chilean youth.

Pathways across tested models	Estimate	Std. error	*Z*	*p*	*CI* lower	*CI* upper	Std. L.V.
*Model 1*							
a	1,075***	0.135	7.974	0.001	0.811	1.339	0.792
b	0,425**	0.146	2.911	0.004	0.139	0.711	0.325
c’	0,899***	0.187	4.818	0.001	0.533	1.264	0.507
d	0.109	0.067	1.617	0.106	−0.023	0.241	0.366
e	1.123	0.657	1.709	0.087	−0.165	2.411	0.188
ab	0,457**	0.163	2.805	0.005	0.138	0.776	0.257
de	0,122*	0.048	2.547	0.011	0.028	0.216	0.069
Total (c)	1,477***	0.140	10.563	0.001	1.203	1.752	0.833
*Model 2*							
a	1,303***	0.152	8.597	0.001	1.006	1.600	0.858
b	0,442*	0.188	2.355	0.019	0.074	0.810	0.335
c’	0,925**	0.272	3.399	0.001	0.392	1.459	0.462
d	0,165*	0.083	1.987	0.047	0.020	0.328	0.409
e	1,147*	0.580	1.978	0.048	0.011	2.283	0.232
ab	0,576*	0.257	2.237	0.025	0.071	1.081	0.288
de	0,190**	0.064	2.959	0.003	0.064	0.315	0.095
Total (c)	1,691***	0.160	10.555	0.001	1.377	2.005	0.844
*Model 3*							
a	1,431***	0.144	9.949	0.001	1.149	1.713	0.893
b	0,503*	0.217	2.322	0.020	0.078	0.929	0.393
c’	0,830*	0.327	2.542	0.011	0.190	1.470	0.404
d	0.159	0.082	1.940	0.052	−0.002	0.319	0.399
e	1.198	0.617	1.941	0.052	−0.012	2.407	0.232
ab	0,721*	0.317	2.271	0.023	0.099	1.343	0.351
de	0,190**	0.065	2.930	0.003	0.063	0.318	0.093
Total (c)	1,741***	0.164	10.629	0.001	1.420	2.062	0.848

*N* = 261; Std. Error, standardised error; Z = Z-value, *p* = value of *p*, Std. L.V. = Standardised Latent Estimate of the Pathway. The term c’ represents the direct effect of Positive Identity on Psychological Wellbeing, whereas ab and de represent mediated indirect effects of Positive Identity on Psychological Wellbeing through Confidence and Character, respectively. ab = Confidence Regressed on Positive Identity and Confidence mediating this relationship on Psychological Wellbeing, de = Character regressed on Positive Identity and Character mediating this relationship on Psychological Wellbeing. Total (c) represents the total effect of Positive Identity on Psychological Wellbeing, which is c’ + ab + de. 95% CIs are reported. *p < 0.05; **p < 0.01; ***p < 0.001.

## Discussion

The results highlight the relevance of studying Latin-American adolescents’ wellbeing in times of COVID-19, as ‘Young Chileans’ Positive Identity significantly predicted their Psychological Wellbeing, thus providing support for H1. Simultaneously, this relationship was mediated by both their level of Confidence and Character, which consequently supported H2 and H3. In the study, positive Identity yielded the highest standardised beta coefficients, followed by Confidence and Character, which is consistent with previous cross-national PYD research (e.g. Dimitrova et al., 2021) and incipient research in the Chilean population conducted by Vera-Bachmann et al. (2020) with the Scale for the Assessment of Developmental Assets in the Neighborhood (SADAN, Oliva et al., 2012). This questionnaire appraises adolescents’ perceptions regarding different resources in the neighbourhood favouring adolescent wellbeing, comprising a five-factor structure: Security, Social control, Support and Empowerment, Youth Activities and Attachment to the neighbourhood. In Vera-Bachmann et al., SADAN proved invariant across genders and educational strata up to the metric level, and it presented scalar invariance regarding age (Putnick and Bornstein, 2016, for a review on measurement invariance), showing the highest factor loadings on Attachment to the neighbourhood, Support and Empowerment and Security, portraying the first mostly internal assets, whereas the second and third mostly external assets. The previous is congruent with the results of the present study regarding the explanatory role of internal assets, as they corroborated the importance of internally driven resources and competence (i.e. trait level) on psychological wellbeing.

Moreover, although the establishment of cultural invariance of the 7Cs and valid PYD measures to test PYD indicators has been claimed in Latin America for widespread applications (Manrique-Millones et al., 2021), researchers cannot safely conduct cross-cultural comparisons, nor is it reasonable to develop and provide valid interpretations from an instrument without having shown that the same underlying construct is being measured across populations (Ziegler and Bensch, 2013). For example, sample bias may arise from a lack of comparability or even minor differences in sample characteristics between the source and target culture samples (Greiff and Iliescu, 2017). The latter supports forthcoming PYD research with Chilean participants, which may be of value to local interdisciplinary practitioners working with emerging adults on behavioural management and fostering a positive outlook and thriving.

A potential sociocultural explanation for the results obtained from the proven model is that “Feeling good about yourself” (i.e. Positive Identity, Dimitrova et al., 2021, p. 25) is rooted in Chilean Youth, given the high individualism enforced since military coup (1973) and further extreme neoliberalism conceived in a dictatorship, which was mostly unaltered after the return to democracy in the country. For instance, basic services are mostly privatised in Chile, such as health, education and the pension scheme, which, together with rises in the cost of living and transportation, affected youth, the elderly and other socially disadvantaged groups, leading to riots, social discomfort and massive manifestations across the country in October 2019, into what it was later locally known as the “18-O.”

The previous account is congruent with the transfer of Chile from Latin America’s shared cultural values to West and South Asia’s shared values in the Inglehart-Welzel World Cultural Map 2022 (Haerpfer et al., 2022; The Inglehart-Welzel World Cultural Map - World Values Survey 7, 2022). For instance, when the last wave of the world values survey (2022) is compared with the previous iterations from the longitudinal study (2014, 2008, 1996), a shift in Chilean culture from traditional values to secular-rational values can be traced (i.e. less emphasis on religion, family values and authority). Although this change preliminarily should be of no concern, it is tied to a setback from an incipient culture based on self-expression after the return to democracy (in 1996, 2008 and 2014’s waves) to a culture in which survival is pivotal (i.e. economic and physical security needs are prioritised, ethnocentric look, low levels of trust and lack of tolerance).

Of course, the increasing levels of individualism tied with high social insecurity in Chilean society permeates youth differently than other age strata. In relation to this, Gabriel Salazar, 2006’s national prize of history, refers to the 18-O surge and the role of youth:

All that was missing was a spark (any spark) that would make everything explode, twitching the skin of Chile’s adolescents, who have been showing more historical sensitivity and political irritability than any other sector of society. That spark came with the rise of the subway and the repression that followed the movement for mass evasion (Salazar, 2019, para 15).

In addition, alternative models tested through SEM and multiple regression analyses showed that Connection was an alternatively important predictor for Psychological Wellbeing and that Caring did not exert an important role. For instance, when the Chilean data were analysed through multiple regression analyses, Positive Identity, Confidence, Character and Connection, altogether explained up to 61% of Psychological Wellbeing variance (Pérez-Díaz, 2022). As for mediation, Connection was not a significant mediator of the relationship between Positive Identity and Psychological Wellbeing in the study.

The most important limitation of the research is that causation cannot be fully established from mediation analyses conducted with cross-sectional data, as other confounders may exert a role on psychological wellbeing, thus restricting the generalizability of our findings (Maxwell and Cole, 2017). However, it is still possible to conduct theory-driven mediation analysis with cross-sectional research when the independent variable is clearly defined as a precedent (Fairchild and Mcdaniel, 2017), and as we argue more so when providing evidence of a double mediation, adding precision and enhancing replication (Agler and De Boeck, 2017). In our case, Positive Identity emerges from developmental processes which cannot be circumscribed to a state. Hence, the independent variable can be comprehended as a trait, and the same rationale applies to the mediators, Confidence and Character. Therefore, the study’s only “state” variable corresponded to psychological wellbeing, which is theorised as the criterion in the tested mediation models. A second limitation of the more advanced models tested in the research, especially from the last model (m2.2), is that there was a substantial number of cross-loadings at the item level between the Confidence and Character factors from the 5Cs model. This may be evidence of the PYD latent variable affecting Psychological Wellbeing. A third limitation pertains to the recommended sample size in SEM, which ought to increase as the mediation modelling becomes more complex and includes more parameters. For instance, Wolf et al. (2013) suggest the sample size in mediation models to be 180–450, depending on the magnitude of structural parameters and total variance explained. Thus, the current study could be slightly undersampled. Moreover, it is expected that future research with Latin-American samples elucidates more precisely the different paths in which Positive Identity, Confidence, Character and potentially Connection predict the variability of other salient wellbeing measures, such as eudaimonic wellbeing, despite these mostly sharing the same factorial space (Keyes, 2005).

## Conclusion

The results supported a partial double-mediation model in which Confidence and Character positively mediated the relationship between Positive Identity and Psychological Wellbeing. Other competence from the 5Cs framework did not significantly influence Psychological Wellbeing through mediation or moderation. However, Connection remains a predictor potentially interesting to be tested on other relevant psychological criteria through mediation and moderation analyses.

## Data availability statement

The datasets presented in this study can be found in online repositories. The names of the repository/repositories and accession number(s) can be found at: https://data.mendeley.com/datasets/76nwjf62kk.

## Ethics statement

The studies involving human participants were reviewed and approved by the University of Bergen, Norway, dated June 24th, 2019, with reference number 612969. In addition, local ethics approval was obtained in Chile on June 25th, 2020, from the Vice-rectory of Research, Development and Artistic Creation (VIDCA)-Ethics and Bioethics Committee of the Austral University of Chile, and their subcommittee on Bioethics in Human Research. The patients/participants provided their written informed consent to participate in this study.

## Author contributions

PP-D contributed to the conception and design of the study, organised the database, performed the statistical analysis, and wrote the first draft of the manuscript. SV, MP, and NW wrote sections of the manuscript. All authors contributed to manuscript revision, read and approved the submitted version.

## Funding

The research was funded by the Faculty of Psychology of the University of Bergen, Norway.

## Conflict of interest

The authors declare that the research was conducted in the absence of any commercial or financial relationships that could be construed as a potential conflict of interest.

## Publisher’s note

All claims expressed in this article are solely those of the authors and do not necessarily represent those of their affiliated organizations, or those of the publisher, the editors and the reviewers. Any product that may be evaluated in this article, or claim that may be made by its manufacturer, is not guaranteed or endorsed by the publisher.
